# Laboratory Markers in the Management of Pediatric Polytrauma: Current Role and Areas of Future Research

**DOI:** 10.3389/fped.2021.622753

**Published:** 2021-03-16

**Authors:** Birte Weber, Ina Lackner, Christian Karl Braun, Miriam Kalbitz, Markus Huber-Lang, Jochen Pressmar

**Affiliations:** ^1^Department of Traumatology, Hand-, Plastic- and Reconstructive Surgery, Center of Surgery, University of Ulm, Ulm, Germany; ^2^Institute of Clinical and Experimental Trauma-Immunology, University Hospital of Ulm, Ulm, Germany; ^3^Department of Pediatrics, University Medical Center Ulm, Ulm, Germany

**Keywords:** organ injury, biomarker, emergency room management, laboratory parameters, coagulation, inflammation, acid-base balance

## Abstract

Severe trauma is the most common cause of mortality in children and is associated with a high socioeconomic burden. The most frequently injured organs in children are the head and thorax, followed by the extremities and by abdominal injuries. The efficient and early assessment and management of these injuries is essential to improve patients' outcome. Physical examination as well as imaging techniques like ultrasound, X-ray and computer tomography are crucial for a valid early diagnosis. Furthermore, laboratory analyses constitute additional helpful tools for the detection and monitoring of pediatric injuries. Specific inflammatory markers correlate with post-traumatic complications, including the development of multiple organ failure. Other laboratory parameters, including lactate concentration, coagulation parameters and markers of organ injury, represent further clinical tools to identify trauma-induced disorders. In this review, we outline and evaluate specific biomarkers for inflammation, acid-base balance, blood coagulation and organ damage following pediatric polytrauma. The early use of relevant laboratory markers may assist decision making on imaging tools, thus contributing to minimize radiation-induced long-term consequences, while improving the outcome of children with multiple trauma.

## Introduction

Multiple trauma is the leading cause of mortality in both adolescents and pediatric patients (1, 2). In most of the recently published studies (particularly in Germany and other high income countries) road traffic accidents are the leading cause of injuries in children, followed by falls from heights (3–6). Motor vehicle crashes account for 78% of severe injuries in children compared to at least 63% in adults (3). In adults, the term “multiple trauma” describes several injuries suffered simultaneously in different parts of the body, whereby at least one injury or the combination of several injuries is life-threatening (7, 8). The injury severity is reflected by the Injury Severity Score (ISS). The ISS is calculated based on the Abbreviated Injury Scale (AIS) and considers the three most severely injured body regions (9). With regard to the ISS definition, “multiple trauma” is defined as an ISS ≥16. This definition is also validated for pediatric polytrauma (4, 10).

The trauma mechanism in children frequently differs from those of adults. The small body height and weight and the special body proportion and constitution determinate injury severity in children. As in adults, male children are more frequently affected by multiple trauma than females (3, 11). This distribution is also observed in infants and toddlers (11). The injury pattern after polytrauma in children is strongly age dependent. Up to the age of schoolchildren, traumatic brain injury (TBI) is the most common trauma consequence. The extremities, chest and abdomen are more frequently affected in older children (11). In particular, chest and brain trauma are predictive for the outcome of pediatric polytrauma patients (12). Because the pediatric chest is more elastic, children display an increased risk for intrathoracic organ damage compared to adults (5). Additionally, lung and/or cardiac contusion can be present in pediatric patients without any external signs on the thoracic wall (6, 13, 14). The surgical treatment strategy of extremity fractures in children differs in comparison to those of adults. While adults are more frequently treated following the Damage Control Orthopedic (DCO) concept [60.3%], fractures in children are stabilized according to the Early Total Care (ETC) principles (49.4%) (15). The incidence of multi-organ failure (MOF) is also described as age-dependent: Younger children less frequently develop MOF compared with older children (16–18). In comparison to adults, late lethality (>24 h) is higher in children, whereas no significant differences were observed in the early phase after trauma (3, 4).

Pediatric polytrauma patients should be treated in a specialized trauma center with an adequate infrastructure and experience in the management of complex injuries (16, 19). Because of differences in anatomy and physiology, pediatric polytrauma patients require early emergency diagnostics adjusted to age-specific variations. Defining appropriate reference values for laboratory tests as well as the interpretation of imaging are needed and are still a matter of debate. Severely injured pediatric patients are rare even in specialized trauma centers. Therefore, physicians involved in diagnostic procedures and management in the emergency room of severely injured children need to be well-trained and should use laboratory diagnostic tools like biomarkers to reliably confirm their diagnosis, therapy or prognosis (3). Because of the longer life expectancy of children, remaining disabilities after pediatric trauma affect children for their entire life and need to be absolutely avoided. Additionally, the need for further therapy as well as for aftercare poses a high socio-economic burden (20).

Although pediatric polytrauma is an important focus in clinical and experimental research, the limited number of patients and the many ethical hurdles of prospective studies in children has resulted in a lack of systematic studies of multiple injured pediatric patients. Therefore, this review aims to present an overview on the state of the art of the role of laboratory biomarkers in the management of pediatric polytrauma and highlights areas of future research. By the early examination of specific systemic parameters and biomarkers for organ injury, the affected organs could be precisely identified after severe pediatric polytrauma. This early estimation of injury severity and localization of affected organs might encourage the usage of specific diagnostic imaging tools, including whole-body computed tomography (CT), X-ray and ultrasound imaging. As a result, the clinical course of the pediatric patient is improved and at the same time, long-term radiation-induced consequences are limited.

### Acute Systemic Inflammatory Markers

Severe tissue damage after trauma triggers the immediate activation of the innate immune system, resulting in an enhanced systemic inflammatory response (21). The extent of systemic inflammation correlates with the injury severity (22, 23). In the clinical setting, it is useful to apply these inflammatory mediators as prognostic surrogates and to define high-risk groups as well as to identify a risk-adapted therapy.

Currently, various inflammatory systemic mediators are clinically used for the early emergency diagnostics of pediatric trauma. The C-reactive protein (CRP) and the number of leukocytes are clinically relevant systemic inflammatory markers, which are commonly used in pediatric patients. Interestingly, a correlation between initial CRP (first 3 hospital days) and the injury severity was described (24). Furthermore, a correlation between the CRP, blood glucose level and mortality rate of children with severe injuries was presented in this retrospective study of 42 trauma patients (mean age 8.0) Remarkably, these parameters were further associated with a prolonged hospital stay (24). Therefore, CRP might be a useful diagnostic biomarker which should be considered for early emergency diagnostics after pediatric trauma. Besides the prognostic value of CRP after trauma, increased CRP levels in febrile children in the emergency department should also initiate further infection diagnostics such as blood culture and smear tests. Furthermore, the Pediatric Early Warning Score (PEWS) and National Institute for Health and Care Excellence (NICE) or the Liverpool quick Sequential Organ Failure Assessment (LqSOFA) (25) are applied in children with acute febrile illness in the pediatric emergency department in order to identify life-threatening infection.

Moreover, in the early inflammatory phase, interleukin (IL)-6 and IL-8 are also used in pediatric trauma care. Compared to adults, the innate immune system is not fully matured in children and the pro-inflammatory cytokine production is, therefore, less pronounced (26, 27). The complexity of the pediatric immune reaction is represented by the fact that a strong release of pro-inflammatory cytokines from macrophages is accompanied by a high production of anti-inflammatory cytokines, including IL-10 (28, 29). Other studies demonstrated an increase of IL-6 and IL-8 in the early post-traumatic phase (30, 31). While the prognostic benefit of IL-6 is well-known in adults (32, 33), the discussion is still ongoing in the case of children. Andruszkow et al. described in 2014 a significant correlation between increased IL-6 during the first 2 days after trauma and MOF development (34). By contrast, our group recently observed no correlation between IL-6 and organ failure in 88 polytraumatized pediatric patients (4). Ozturk et al. observed a significant difference only in the survival of severely injured children (forty-seven children (37 boys, 10 girls) presenting with blunt trauma),with regard to IL-8, whereas IL-6 and the early cytokines tumor necrosis factor (TNF) and IL-1β displayed no association with the survival rate (35). Consequently, the early evaluation of systemic inflammatory cytokines after pediatric trauma might be a useful tool in emergency diagnostics for the adequate estimation of systemic inflammation. However, the complexity of the pediatric immune reaction should be carefully considered and the analysis of further inflammatory parameters like IL-1β and IL-12p70 might promote the adequate assessment of trauma-induced inflammation in pediatric patients.

Another possible valid indicator for pediatric trauma is procalcitonin (PCT), because a strong correlation between PCT and the injury severity was shown in adults. Moreover, PCT correlated with the development of post-traumatic sepsis (36–38) and might be a strong predictor for the development of MOF after trauma. Therefore, PCT appeared to be a reliable prognostic marker after trauma in adults. In children, there are also studies describing PCT as an independent predictor for the development of sepsis and of the systemic inflammatory response syndrome (SIRS) after trauma. Moreover, a correlation of plasma PCT and the injury severity in children was observed. Therefore, blood samples of 30 children with acute trauma were investigated, in which 23% developed sepsis and the PCT peaked at day 2. PCT at day 2 was an independent predictor of the development of sepsis in children (39, 40).

**Figure 1:**
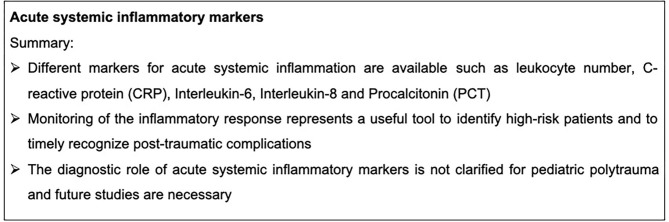
Summary box acute systemic inflammatory markers.

Thermal injuries are major causes of morbidity and mortality. Patients frequently suffer burns combined with trauma, which is also described as “two-hit” phenomena of injury, resulting in a higher morbidity of the affected patients because of synergistic detrimental effects (41, 42). Burn injuries are severe concomitants after explosions or motor vehicle crashes, affecting both adults and children (43). Burn injuries are associated with a massive inflammatory response, which appeared to be similar between adults and children. Worthy of note is that IL-6 and IL-10 blood plasma levels were significantly reduced early after burn injury in children. In this study 25 adults and 24 children were enrolled who survived a flame burn covering more than 20% of total body surface area and cytokine levels were measured within the first week after trauma (44). These parameters and their ratio were associated with a poor outcome in pediatric trauma patients (22, 23). However, the role of age in the post-traumatic inflammatory response in children remains unknown. Following pediatric burn injury, neither CRP nor PCT necessarily correlated with an increased mortality (45). Consequently, further studies investigating the systemic inflammatory profile after burn injury combined with severe trauma in children are necessary. Markers for acute systemic inflammation are summarized in Figure 1.

### Acid-Base Balance

**Figure 2:**
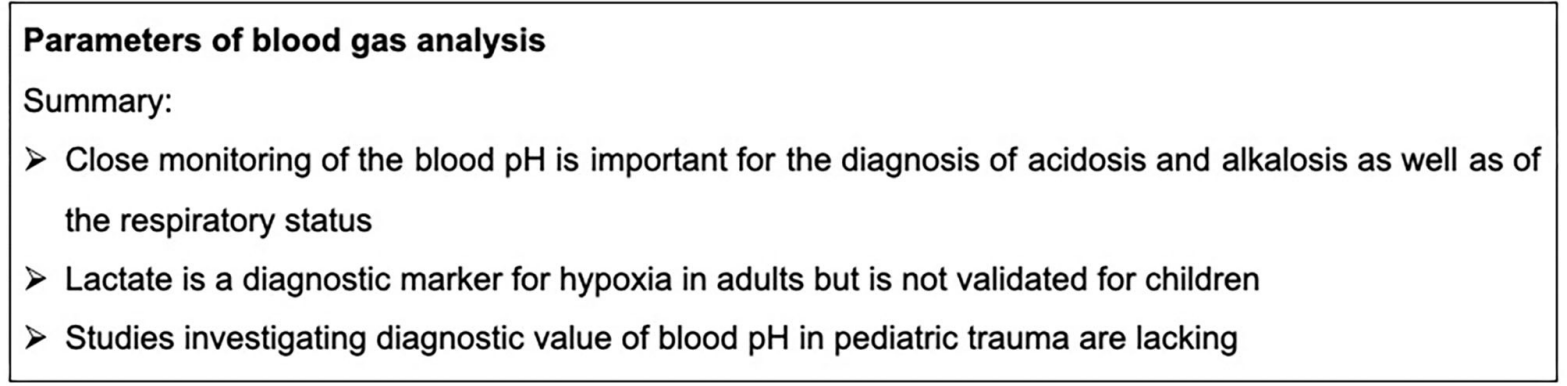
Summary box parameters of blood gas analysis.

The acid-base balance is important for the clinical management of severely injured children. It is monitored by conducting a blood gas analysis (BGA), which is routinely performed. One prognostic marker for an imbalanced acid-base equilibrium is lactate. Lactate is produced during anaerobic metabolism and is an established marker for tissue hypoxia. However, the prognostic validity of lactate for pediatric trauma is ambiguous and it is currently unclear how lactate production in children differs from adults after severe trauma (46). A systematic review of Lawton et al. showed a strong correlation between initial high lactate levels and mortality after multiple trauma in adults (46). In another study, post-traumatic lactate levels were measured in more than 210 injured children (47). In this study, a correlation between high lactate levels and the injury severity was demonstrated, which was further confirmed by other studies (48). Our group recently described high levels of lactate in severely injured children, but no correlation with the injury severity (4). Interestingly, Fu et al. reported a high prognostic importance of lactate in the case of pediatric TBI investigated in 213 with an GCS < 13 (49). Nevertheless, the base deficit as a prognostic marker for pediatric trauma is strongly debated. Some studies propose the base deficit as a prognostic marker of the injury severity and mortality (50, 51), but Levy et al. indicated an only weak predictive value of the base deficit (52). Parameters for blood gas analysis are summarized in Figure 2.

### Coagulation Tests

Acute trauma-induced coagulopathy (ATIC) is a severe consequence in multiple injured patients as well as in children resulting in a high mortality rate after trauma (53, 54). The incidence of ATIC after severe trauma varies between 20–70%, which might be due to different international ATIC definitions (53–56). Additionally, post-traumatic consumption of coagulation factors and hypercoagulation with microthrombosis frequently occur in children after severe trauma (57, 58).

The hemostasis system of children significantly differs from that of adults, whereby the clinical interpretation of laboratory routine parameters might be challenging (59, 60). Additionally, there is a clear lack of systematic studies investigating ATIC in children, which are needed to estimate its prognostic role as well as to define diagnostic and therapeutic strategies for ATIC treatment. Currently, the evaluation of the classical coagulation parameters represents an important tool for the diagnosis of severely injured children.

Most relevant parameters for the detection of ATIC are the prothrombin time, the partial thromboplastin time (pPTT) as a marker of the plasmatic coagulation, the fibrinogen, the fibrin cleavage products and the thrombocytes as parameter of the cellular coagulation. The advantages of these markers are their ubiquitous availability as well as the relatively reliable interpretation. Nevertheless, the coagulation system is strongly dependent on patient age. Particularly newborns and toddlers present a lack of vitamin K dependent coagulation factors, less fibrinogen and reduced thrombocytes, displaying a higher risk for coagulopathy compared to older children and adults (59). The international normalized ratio (INR, >1.2–1.5) is frequently used to define coagulopathy in severely injured children. An increase of the INR is likewise associated with an increased mortality investigated in a cohort analysis of 744 patients with an age < 18 years (early coagulopathy was observed in 27%) (54, 55). Furthermore, an increased aPTT and a reduced platelet count on hospital admission also correlate with increased mortality in a cohort of 91 injured children [33 children showed coagulopathy at admission, seven did not survive (21%)] (61).

The international society of thrombosis and hemostasis (ISTH) developed a score, which summarizes the INR, plasma fibrinogen, d-dimer/fibrin cleavage products and the amount of thrombocytes to diagnose a disseminated intravascular coagulopathy (DIC) (62). This score was also validated in pediatric patients with sepsis and/or circulatory shock correlating with the mortality of these children (58, 63). Therefore, this DIC-score of the ISTH may be a helpful tool in the diagnostic of ATIC in severely injured children.

**Figure 3:**
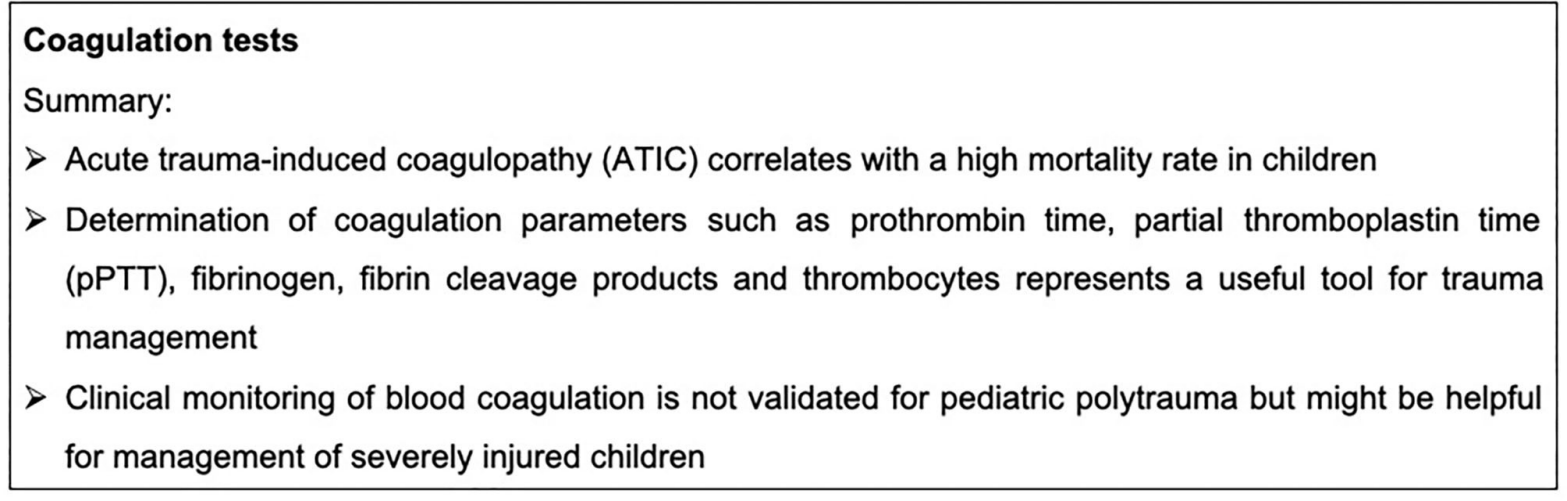
Summary box coagulation tests.

Currently, the so-called “viscoelastic measurement” thromboelastography (ROTEM®) is considered a rather reliable point-of-care monitoring of acute coagulopathies in the emergency room and intensive care unit (ICU). In addition to the assessment of initial coagulation, it is also a suitable tool to immediately evaluate therapeutic success. Although a wide range of studies exists in adults, there remains a lack of literature about the usefulness of thromboelastography in children. In one case report of a 7-year-old boy, the successful use of thromboelastography as a monitoring tool of the fibrinogen concentration was described (64). Thromboelastic measurements were also described as controlling the transfusion of fresh frozen plasma in severely injured patients more adequately than the INR (65). The future role of thromboelastic measurements in the case of ATIC children is still unclear and requires further investigation. Coagulation tests are summarized in Figure 3.

### Markers for Organ Injury

Severe trauma frequently affects the musculoskeletal system as well as the internal organs. On the one hand, there are direct blunt or penetrating injuries, particularly of the abdomen or chest. These injuries directly damage the contained organs. On the other hand, the early inflammatory response after trauma may induce a secondary damage of the organs. In severely injured children, organ damage is primarily assessed by various imaging methods, including ultrasound, X-ray, CT and MRI. Additionally, organ damage after trauma is further characterized by the systemic elevation of organ-specific biomarkers. Particularly damage of the heart, kidney and liver are reflected by various well-established laboratory biomarkers. By contrast, brain, lung and spleen damage is more frequently diagnosed by advanced imaging techniques rather than by the assessment of systemic biomarkers. However, the additional evaluation of specific biomarkers for respective organ damage might support and facilitate the early diagnosis in pediatric trauma.

### Markers for Cardiac Injury

Cardiac injuries are frequently recorded after severe multiple trauma and are associated with dysrhythmias, ventricular fibrillation, impaired cardiac function, sudden cardiac arrest and a prolonged ventilation interval as well as by a longer hospital stay of the patients (66–69). Cardiac troponin I is a reliable biomarker for cardiac damage, which is routinely used in clinics for the diagnosis of heart failure in adults (70). Moreover, the elevation of systemic cardiac troponin I is also used in the emergency room diagnostics for the early assessment of myocardial damage after severe trauma in adults. In multiple injured patients, a significant systemic increase of troponin indicates post-traumatic cardiac damage, which is further associated with an increased mortality as well as with an enhanced demand for catecholamines (32). The diagnostic and prognostic value of systemic troponin in 88 severely injured children was recently described by our group (4). In this study, we measured enhanced systemic levels of troponin in children at hospital admission, which correlated with systemic IL-6 and creatine kinase levels. Children with an initial troponin >14 ng/ml required significantly longer intensive care. In addition, the young patients who were diagnosed with lung contusion or MOF or who died after multiple trauma presented significantly higher initial systemic troponin concentrations compared to those without post-traumatic complications. With regard to these observations, systemic cardiac troponin might also be a reliable prognostic marker for cardiac damage in severely injured children (4). Nevertheless, the age of the children should be carefully considered when analyzing systemic troponin levels: Preterm infants have tenfold higher baseline TnT levels compared to newborns (71). Interestingly, myocardial contusion or any macroscopic tissue damage of the heart was not regularly observed in an autoptic study of 282 pediatric polytrauma patients (age < 16 years) (72). This finding is in accordance with an experimental model of multiple trauma and hemorrhagic shock in mice which did not present localized tissue damage of the heart, although a significant increase of troponin was described (73). These observations might indicate functional, subcellular damage of the cardiomyocytes, which is not detectable in advanced imaging. How troponin is released from morphologically intact cardiomyocytes after trauma remains unknown (74). Furthermore, it remains unclear whether and to what extent cardiomyocytes are able to regenerate. For example, complete recovery of cardiac function in newborns after myocardial infarction was observed (75). Furthermore, in rodents, cardiac regeneration after myocardial injury was described up to the first 7 days post-birth by hyperplastic growth (76).

In addition to troponin, the early biomarker heart fatty acid binding protein (HFABP) is currently used in the preclinic to detect early myocardial damage after trauma (77). In newborn pigs that suffered from asphyxia, hemorrhage and underwent cardiopulmonary resuscitation, a systemic increase of troponin I as well as of HFABP was observed 4 h after trauma. In children with congenital heart failure, ischemia or kidney injuries, HFABP is described as a reliable biomarker during pediatric age (78–80). To answer the question whether HFABP should be included in the laboratory diagnostic of pediatric polytrauma patients, further studies are necessary.

**Figure 4:**
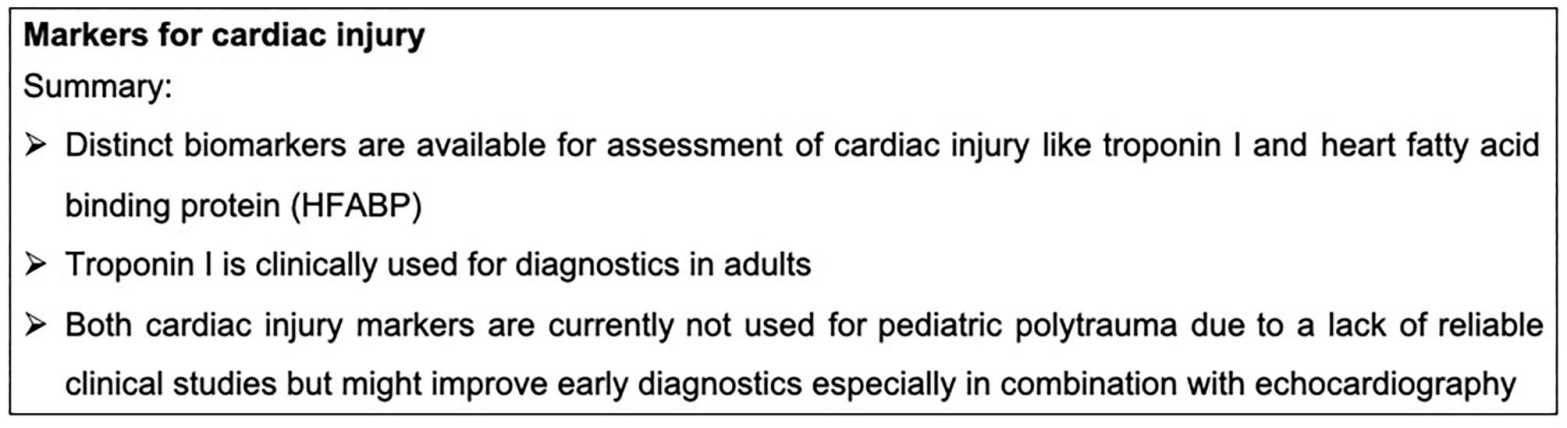
Summary box markers for cardiac injury.

Apart from direct mechanical cardiac damage, the development of post-traumatic cardiomyopathy is one example for secondary organ damage, which is well-described during sepsis as well as after trauma (81–83). Secondary cardiac damage after trauma, including functional and structural alterations, was linked to pro-inflammatory cytokines (84, 85), local damaging reactive oxygen species (ROS) (86) as well as danger-associated molecular patterns (DAMPs) (87, 88). Furthermore, local changes in the complement receptor expression of cardiomyocytes as well as alterations of the electromechanical signaling via gap junction endocytosis were observed after trauma and were further associated with the development of post-traumatic cardiomyopathy (73, 77, 89, 90). Markers for cardiac injury are summarized in Figure 4.

### Markers for Kidney Injury

In children, 10% of blunt abdominal trauma events demonstrated a kidney lesion (91). Children are more susceptible to kidney-associated trauma consequences because of the small proportion of retroperitoneal and abdominal fat tissue, the kidney size, the weak expression of muscles, the elastic ribs as well as the kidney localization (92, 93). Moreover, children more frequently display some anatomical anomalies, including ureteropelvic junction obstruction and a horseshoe kidney. These anomalies are responsible for an increased susceptibility for traumatic kidney lesions (93, 94). Acute trauma-related kidney injury was associated with increased mortality in 88 children with an age between 0 and 20 years (95). Currently, the diagnostic method of choice for the examination of traumatic kidney injury in children is ultrasound. In case of severe injury, the application of contrast agents and imaging via CT scan could be considered. MRI could also be used for diagnosis in young children (93, 96). An apparent warning signal for traumatic kidney injury is macrohematuria, which needs to be controlled via advanced imaging (97). Furthermore, micro-hematuria following pediatric blunt trauma which occurred in 19% of 1059 children with blunt abdominal trauma did not impact the management in 78% (98). However, a retrospective review of 655 children aged 0-16 years with motor vehicle accident revealed a positive predictive value of 39% and a negative predictive value of 87%. In this report micro-hematuria was further associated with increased hospital stay, surgical interventions and admission to intensive care unit (99). Accordingly, the diagnostic role of a screening urine dipstick has low sensitivity and specificity but is a useful and inexpensive screening tool.

**Figure 5:**
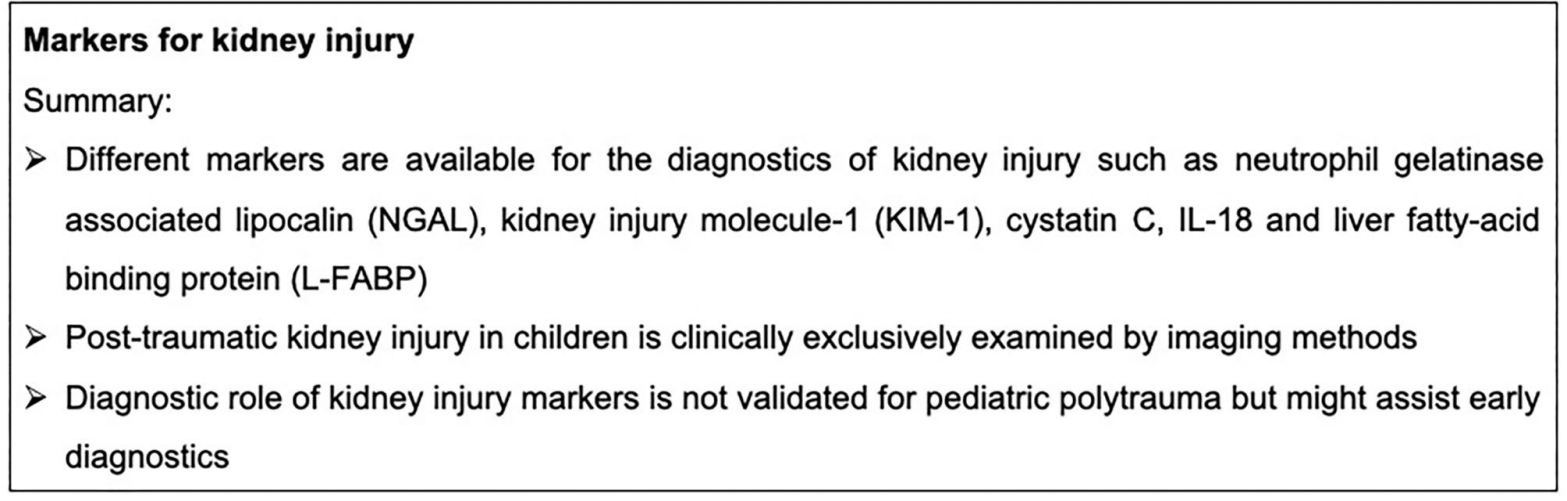
Summary box markers for kidney injury.

Of note, severe tissue injury can also result in development of remote trauma-related acute kidney injury (TRAKI) (100) even in absence of any primary kidney injury. Direct and indirect kidney damage could be assessed by specific biomarkers. A classical systemic kidney marker is creatinine. However, this laboratory parameter appears to be unreliable in the emergency diagnosis of traumatic kidney injury, because changes in creatinine concentrations occurs only when kidney function is reduced to more than 50% (101, 102). Furthermore, the creatinine concentration is significantly influenced by a skeletal muscle trauma. Additional biomarkers have been recently discussed as markers for traumatic kidney injury, including neutrophil gelatinase associated lipocalin (NGAL), kidney injury molecule-1 (KIM-1), cystatin C, IL-18 and liver fatty-acid binding protein (L-FABP) (102). NGAL appeared to be a promising biomarker for traumatic kidney injury in multiple injured patients, with it being a well-established marker for the development of post-traumatic kidney dysfunction (103, 104). The diagnostic role of NGAL after pediatric trauma is currently not described. However, after pediatric burn injury, NGAL serum concentrations as well as its urine levels correlated with the development of acute kidney injury. Twenty-two children were enrolled and six (27%) of them developed AKI within the first 48 h after injury. Moreover, NGAL correlated with CRP and PCT as well as with the urine albumin and creatinine concentrations after burn injury in children (105). Nevertheless, NGAL is not frequently available in the emergency routine diagnostic and its prognostic role in the emergency case after pediatric trauma remains unknown. Markers for kidney injury are summarized in Figure 5.

### Markers for Liver Injury

In pediatric trauma, the kidneys and the liver are the most commonly injured abdominal organs. Particularly hemodynamic relevant bleeding is responsible for a high mortality after traumatic liver injuries (106, 107). In total, 4% of pediatric trauma cases display a detectable liver injury (108). Traffic accidents are a common mechanism of traumatic liver damage followed by falls from a height (109). Currently, trauma-induced liver injury in pediatric patients is mainly diagnosed by imaging methods. The diagnosis of traumatic liver injury is commonly based on CT scans, which, however, should be considered carefully in pediatric age. Therefore, the first step to diagnose liver damage in severely injured children is the performance of an ultrasound. When there are any suspicious findings in the initial ultrasound assessment, a CT scan should be considered. The radiological extent of liver injury does not correlate directly with the urgency for an emergency operation (110). A useful tool in the emergency room for diagnostic of abdominal bleeding and organ injury is the so-called FAST-ultrasound technique (Focused Assessment with Sonography for Trauma). Nevertheless, this commonly applied screening should be always combined with a careful clinical examination (111, 112).

**Figure 6:**
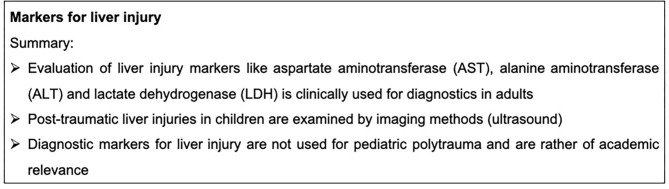
Summary box markers for liver injury.

In addition to imaging techniques, traumatic liver injury could be further detected by specific systemic biomarkers. In adults with severe liver trauma, aspartate aminotransferase (AST), alanine aminotransferase (ALT) lactate dehydrogenase (LDH), high INR values and low fibrinogen levels at hospital admission are associated with a high mortality. (113). As in adults, the systemic increase of ALT after pediatric abdominal trauma represents a predictive value of a clinically relevant liver injury. In a study with 205 pediatric trauma patients, 87 children displayed a significant elevation of one or both transaminases (43% AST, 35% ALT). Nevertheless, only transaminases of >400 U/l were associated with a degree of liver injury identifiable by abdominal imaging. In total, 67% of the children with AST levels over 400 U/l and 78% with ALT levels >400 U/l were found to have a gradable liver injury (108). Additionally, an extremely rapid and high rise of ALT levels were associated with severe liver injuries in adults (114). A negative initial ALT in hemodynamically stable children does not justify diagnostics via a CT scan (115). In a survey report of Swiss surgeons, 58% were convinced that pediatric patients do not require a CT scan despite anomalies in the initial examination or ultrasound. Interestingly, they decided to conduct further imaging when the results of the ultrasound were suspicious but did not base their decision on laboratory liver function tests (114). Markers for lung injury are summarized in Figure 6.

### Markers for Traumatic Brain Injury

TBI is the leading cause of mortality among adolescents and children (116). Children surviving TBI sustain moderate to severe injuries and frequently suffer from long-term disabilities (117). Falls, sports- and recreation-related blunt force trauma and motor vehicle accidents are the leading causes of pediatric TBI. Worthy of note is also that child abuse can be the underlying cause of severe TBI. Acceleration-deceleration injuries can result in diffuse axonal injury (DAI) (117). DAI refers to extensive structural damage of neuronal tissue because of abrupt stretching, twisting and/or shearing of axons induced by mechanical blows to the head (118). Particularly in children, TBI has detrimental long-term consequences, including the development of critical neurobehavioral functions. Moreover, the recovery from TBI in the developing brain differs greatly from that of the mature adult brain (119). For the management of pediatric TBI, neuroimaging is commonly used to improve clinical care and management of children (120). For an adequate estimation of brain injury, CT is applied post trauma for the immediate detection of extra-axial hemorrhage, acute hydrocephalus, fractures and other intracranial lesions (121). Moreover, MRI is a very sensitive technique for the detection of intra-parenchymal lesions. Advanced MRI techniques have been established during recent decades for the identification of sequelae as well as for management decisions of pediatric TBI (122).

**Figure 7:**
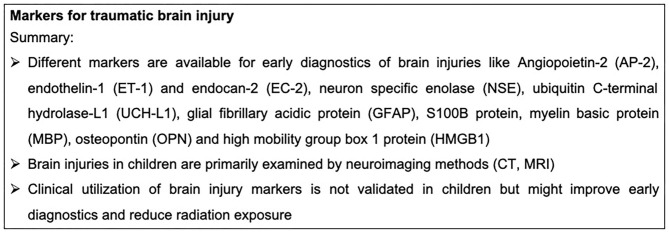
Summary box markers for traumatic brain injury.

In addition to neuroimaging techniques, plasma biomarkers might be a reliable tool for the clinical assessment of pediatric TBI. Angiopoietin-2 (AP-2), endothelin-1 (ET-1) and endocan-2 (EC-2) were described to be elevated after TBI in children (28 children hospitalized with mild, moderate, and severe TBI), correlating with both their GCS and ISS (123). Moreover, the neuronal cell body injury markers neuron specific enolase (NSE) and ubiquitin C-terminal hydrolase-L1 (UCH-L1) are systemically elevated after pediatric TBI and are considered as predictors for a poor outcome after TBI, which was analyzed by a meta-analysis including 10 studies (124, 125). Noteworthy, UCH-L1 is regarded as a highly sensitive marker for intracranial lesions. It predicts undetected microstructural injuries even in pediatric patients with a normal CT. Moreover, it was shown that UCH-L1 is released together with the astroglial marker glial fibrillary acidic protein (GFAP) after pediatric TBI, correlating with a poor outcome of the children. This study investigated a cohort of 45 children with the clinical diagnosis of TBI (GCS 3-15) compared to 40 healthy patients (125). Another astroglial marker is the S100B protein, which is also released after pediatric TBI, and correlates with TBI severity (126, 127). For TBI diagnosis, the combination of CT with systemic S100B protein levels has also been suggested (127). Additionally, the myelin basic protein (MBP) is systemically increased after TBI in 100 children compared to 64 healthy controls (128). Early hyperglycemia predicts in-hospital mortality in children with moderate to severe TBI (129). Serum lactate is a by-product of anaerobic metabolism and correlates with the injury severity in adult patients after trauma (130). Similarly, the serum lactate levels also correlate with increased in-hospital mortality of children with moderate to severe TBI, as also shown in adults (49). Osteopontin (OPN) is a phosphoprotein which is secreted by macrophages and activated microglia. OPN was found to be systemically enhanced in pediatric TBI, correlating with TBI severity, intracranial lesions and mortality of the children (three to 9 years of age, *n* = 66, GCS </= 8) (131). Interestingly, serum albumin levels could also be considered as predictors for mortality of children (*n* = 213, GCS </=13, 45 died in hospital) with moderate to severe TBI (132). Pediatric TBI is associated with a massive systemic release of inflammatory mediators. Similarly, IL-6 is considered to be a reliable prognostic biomarker for pediatric TBI (123). The administration of 20-hydroxyeicosatetraenoic acid improved the functional outcomes of rats in an experimental pediatric TBI model by decreasing the gene expression of TNF and IL-1β (133). Moreover, microglia/macrophages might also play an important role in the injury mechanisms following pediatric TBI (134). The high mobility group box 1 protein (HMGB1) is a key mediator of neuroinflammation and neurodegeneration in TBI. It was previously shown that HMGB1 is systemically released after experimental pediatric TBI in mice and the inhibition of HMGB1 reduced brain edema and improved short-term spatial memory and motor behavior. However, HMGB1 inhibition did not reduce the severity of evoked seizures or cortical tissue loss in this animal model (135). By contrast little is known about the role of different inflammatory mediators and DAMPs during neuroinflammation following pediatric TBI. Markers for traumatic brain injury are summarized in Figure 7.

### Markers for Lung Injury

A pediatric chest trauma, particularly in combination with other organ damage, including TBI and abdominal lesion, results in an increasing overall mortality after multiple trauma (12). The organs of the thorax are more sensitive to damaging influences in children compared to adults because of the pending ossification of the ribs and the more flexible ribcage (5). In addition to the heart, the lungs are also frequently affected after severe multiple trauma. A lung contusion could lead to impaired oxygenation and subsequently to hypoxemia (13). Pulmonary injury in severely injured children is a common complication and exacerbates the clinical outcome because pulmonary injuries impair ventilation, followed by subsequent lung infections or by a respiratory distress syndrome (13, 136). Following pediatric trauma, lung contusion, rib fractures, pneumothorax/hematothorax or tracheobronchial rupture occur (137). With an occurrence of 36% in injured children, lung contusions are the most frequent consequence of a chest trauma followed by a pneumothorax in 12–22% of the young patients and by rib fractures in 13% of the 33 injured children (age < 18 years) (138, 139). Furthermore, 0.5% of children develop acute respiratory distress syndrome (ARDS) after trauma, which is based on the pulmonary endothelial injury and the epithelial breakdown leading to the subsequent development of alveolar edema (140). Pulmonary edema is associated with increased mortality as described in the context of acute lung injury in adults (141). ARDS-associated mortality arises in 18–35% and occurs mostly in the first week after trauma (142–144). The development of pediatric ARDS is associated with TBI in multiple injured children. ARDS was identified in 0.5% (2660/488,381) of the analysis cohort, with an associated mortality of 18.6% (494/2660) (142).

Currently, in the clinic, pediatric lung injury after severe trauma is diagnosed by imaging techniques. Posterior-anterior X-ray imaging remains one of the basic examination tools to evaluate the consequences of a chest trauma (137). In addition to a lower exposure to radiation, the X-ray imaging has much lower costs compared to CT scans. However, CT should be considered when there are abnormalities in the initial diagnosis (145, 146). This decision should be combined with a clinical evaluation and careful examination. In children, lung ultrasound is frequently used as a diagnostic tool for lung contusion (147, 148). Hypoxia is established in young children as a good predictor of thoracic injury with lung damage, whereas the respiratory rate is only a predictor in adults (138).

Presently, different biomarkers for lung injury are being discussed. The cardiac-specific marker troponin is also considered as a reliable marker of lung contusion after pediatric trauma (149). We recently described an initial troponin T elevation in pediatric trauma patients with lung injury. Furthermore, we observed in 34% of multiple injured children the development of a lung contusion, which was diagnosed by ultrasound and/or CT (4).

In adults, blunt chest trauma results in high serum levels of surfactant protein D (SP-D). A correlation between the SP-D levels and the ISS as well as the development of complications were reported (150). An elevation in serum SP-D-levels also correlated with the mortality of patients receiving mechanical ventilation and was described in the context of obstructive pulmonary disease, pneumonitis and pneumonia (151). Recently, we observed a systemic increase of SP-D after experimental hemorrhagic shock and cardiopulmonary resuscitation in newborn pigs (152). It is therefore tempting to speculate that SP-D might be a reliable biomarker for lung injury after pediatric trauma.

**Figure 8:**
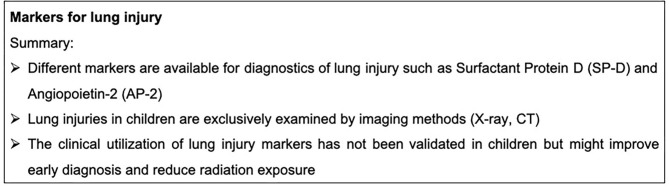
Summary box markers for lung injury.

Furthermore, angiopoietin 2 (AP-2) and the soluble receptor for advanced glycation end products (sRAGE) are discussed as biomarkers for endothelial and pulmonary epithelial damage in pediatric ARDS. AP-2 and sRAGE were higher in survivors compared to non-survivors of pediatric ARDS. Moreover, these biomarkers correlate with the number of critically ill children with non-pulmonary organ failure (153, 154). Additionally, the inflammatory response could be relevant for prognostics in the case of lung injury: IL-6, IL-8, IL-10, IL-18 and TNF-R2 strongly correlated with the overall mortality and the endothelial injury in pediatric patients. Interestingly, both IL-6 and IL-8 displayed a strong correlation with AP-2 (155). Markers for lung injury are summarized in Figure 8.

## Conclusion

The severely injured child remains an interdisciplinary challenge (19). A summary of the respective laboratory markers and imaging tools for the management of pediatric polytrauma are summarized in Supplemental Table 1 and Supplemental Table 2. Although there is an increasing amount of data, there remains a lack of prospective controlled studies to develop guidelines for rapid diagnostics after pediatric trauma. One limitation of the present review is that we only included a selection of references of this wide field. To summarize, many laboratory markers were recently described, which might support the early diagnosis and prognosis of severely injured children. However, for an adequate assessment of the injuries after pediatric trauma, the application of imaging techniques is indispensable and is currently exclusively applied in the clinic. However, the combination of imaging techniques and a reliable prognostic laboratory biomarker could improve the rapid and adequate assessment of pediatric injuries after trauma. Furthermore, early laboratory diagnostics and follow-up measurement could improve the overall outcome and the further clinical process of severely injured children. Moreover, by establishing reliable biomarkers for clinical monitoring in future for pediatric trauma, the exposure of the children to high amounts of radiation might be reduced, preventing radiation-induced long-term consequences.

## Author Contributions

BW, IL, CKB, MK, MH-L, and JP substantially contributed to conception and design and acquisition of data and drafting the article. BW and IL wrote the paper. All authors critically revised the final version of the paper and approved this review to be published.

## Conflict of Interest

The authors declare that the research was conducted in the absence of any commercial or financial relationships that could be construed as a potential conflict of interest.

## Supplementary Material

Supplemental Table 1 Laboratory markers and imaging tools for the management of pediatric polytrauma.

Supplemental Table 2 Laboratory markers in the management of pediatric polytrauma.
