# Supplementary material for: Laboratory Markers in the Management of Pediatric Polytrauma: Current Role and Areas of Future Research
Source: Front Pediatr. 2021 Mar 16;9:622753. doi: 10.3389/fped.2021.622753 (PMC8010656; doi:10.3389/fped.2021.622753)
Supplement: Supplemental Table 1 — Laboratory markers and imaging tools for the management of pediatric polytrauma. [file Table_1.DOCX]

| Biomarker | Results | Imaging |
| --- | --- | --- |
| Emergency room management | | |
|  | No justification to conduct whole-body CT only based on the trauma mechanism (22) | Whole-body CT |
|  | Whole-body CT cannot decrease mortality in comparison to selective CT scans (23) | Selective CT scans of organ regions |
|  | Time consuming, no radiation, no radiological long-term consequences (24–26) | MRI |
|  | Indication: especially in case of intrathoracic organ damage such as lung contusion, pneumothorax or heart injury a diagnostic option (27,28) and FAST | Ultrasound |
| Acute systemic inflammation | | |
| C-reactive protein (CRP) | Correlation with the ISS, the blood glucose and the mortality + associated with prolonged in-hospitalization (40).The correlation with the mortality is still controversial (39,40). |  |
| Procalcitonin (PTC) | Correlation with post-traumatic Sepsis, MOF (multi-organ failure) (41–43) and ISS (44,45) |  |
| Interleukin (IL-) 6/IL-8 | Both are commonly used in the clinic. The prognostic benefit is still controversially discussed in children (9,50). Significant difference exists only in the survival of severely injured children with regard to IL-8, whereas IL-6 and the early cytokines tumor necrosis factor (TNF) and IL-1ß demonstrated no association with the survival (51). Accompanied by IL-10 increase as anti-inflammatory mediators (36,37). |  |
| Acid-base equilibrium (BGA) | | |
| Lactate | Initial lactate correlates with the post-traumatic mortality (52). The correlation with the Injury severity score (ISS) is controversially discussed in children (9,53). Systemic lactate represents a high prognostic value after pediatric traumatic brain injury (55). |  |
| Base deficit | Some studies declare the base deficit as good prognostic value of the ISS and mortality (56,57). Other studies propose a weak predictive value of the base deficit (58). |  |
| pH | Studies in pediatric trauma are still missing. pH should be included in the point of care diagnostics, because its influence on post-traumatic coagulopathy (59). |  |
| Coagulation | | |
| pPTT | Broad availability. Reliable interpretation. pPTT is a marker of plasmatic coagulation and correlates with the mortality (67). |  |
| Fibrinogen | Broad availability. Reliable interpretation. Reliable marker of the fibrin cleavage. Especially newborns and toddlers present a lack of vitamin K-depending coagulation factors, less fibrinogen and reduced thrombocytes, leading to a higher risk of coagulopathy compared to older children and adults (64). |  |
| Thrombocytes | Broad availability. Reliable interpretation. A reduced platelet count at the hospital admission correlates with an increased mortality (67). |  |
| International normalized ratio (INR) | Broad availability. Reliable interpretation. INR is often used to define coagulopathy in injured children. INR is associated with increased mortality in children (61,62). |  |
| DIC-Score | A summarizing score (INR, plasma fibrinogen, d-dimere, thrombocytes), which was validated in pediatric patients with sepsis or shock. Correlation with the mortality (66,69). |  |
| Thromboelastography  (e.g. ROTEM) | Lack of literature about the usefulness of ROTEM in children. One Case report of a 7 year old boy, which describes the successful use of ROTEM as monitoring tool of the fibrinogen concentration (70). |  |
| Cardiac damage | | |
| Troponin | High systemic levels of troponin in children at hospital admission correlates with systemic IL-6 levels and creatine kinase, as well as a significantly longer intensive care (9). Children with lung contusion, MOF or fatal multiple trauma presented exhibited significantly higher initial systemic troponin concentrations (9). The age of the children should be carefully considered in diagnostics via troponin: preterm infants have a tenfold increase in baseline troponin T levels compared to newborns (84). | Echocardiography 🡪 cardiac function |
| Heart-Fatty Acid Binding Protein (HFABP) | HFABP is described as reliable biomarker in pediatric age (89–91). Not routinously used in the clinic so far. | Electrocardiogram 🡪 arrhythmias |
| Kidney injury | | |
| Macro-hematuria | An apparent warning signal for traumatic kidney injury is the macrohematuria which needs to be controlled via imaging (98). | Ultrasound >MRI >CT (95,97) |
| Creatinine | Unreliable in early diagnostics of traumatic kidney injury since changes in creatinine concentrations occurs only if the kidney function is reduced to less than ca. 50% (100,101). Creatinine concentrations depend on musculo-skeletal trauma. |  |
| Neutrophil gelatinase associated lipocalin (NGAL) | NGAL in serum and urine in burned children correlates with the development of acute kidney dysfunction, CRP, PCT and albumin; as well as creatinine concentrations in the urine (102). NGAL is currently not routinely available in the emergency diagnostics. |  |
| Kidney injury molecule-1 (KIM-1), cystatin C, IL-18, liver-fatty acid binding protein (L-FABP) | These proteins are discussed as potential future-biomarkers (101). |  |
| Liver injury/bleeding | | |
| SIPA (shock index pediatric-adjusted) | SIPA was considered as elevated at >1.22 for 4–6.9 year old children, >1.0 for 7–12.9 years of age, and >0.9 for 13–16.9 year old children, whereas the SI (not age-adjusted) was considered elevated if >0.9 (114). With regard to the SIPA index significantly more patients were identified to require additional treatment resources like blood transfusion, operative interventions and ICU care compared to conventional SI (113). | FAST-Ultrasound 🡪 especially in case of hemodynamic relevant bleeding (105,106) 🡪 combined with careful clinical examination (110,111) |
| Aspartate aminotransferase (AST), alanine aminotransferase (ALT) | Transaminases of >400 U/l were associated with a liver injury degree identifiable by abdominal imaging (107). Furthermore, an extremely fast and high rise of ALT levels was associated with severe liver injury in children (115). A negative initial ALT in combination with hemodynamic stable children does not justify diagnostics via CT scan (116).  AST and ALT are considered mainly of academic relevance. | If there are any suspicious findings in the first ultrasound assessment, a CT scan should be considered. Cave: The radiological extent of liver injury does not correlate directly with the urgency of emergency operation (109). |
|  |  | In case of a hemodynamic instable patient, who is not adequately reacting to volume therapy, an emergency laparotomy is indicated (106). |
|  |  | Abdominal bleeding caused by liver injury can be detected by angiography, which additionally have the advantage of an immediate intervention option (embolization)(112) |
| Traumatic Brain Injury (TBI) | | |
| Angiopoietin-2 (AP-2), endothelin-1 (ET-1) and endocan-2 (EC-2) | AP-2, ET-1 and EC-2 were described to be elevated after TBI in children correlating with the corresponding GCS and ISS (125). | CT is used post trauma for the immediate detection of extra-axial hemorrhage, acute hydrocephalus, fractures and other intracranial lesions (123) |
| Neuron specific enolase (NSE) and ubiquitin C-terminal hydrolase-L1 (UCH-L1) | NSE and UCH-L1 are systemically elevated after pediatric TBI and are considered as predictors for a bad outcome after TBI. UCH-L1 is regarded as a highly sensitive marker for intracranial lesions (126,127). | MRI is a very sensitive technique for detection of intra-parenchymal lesions. Advanced MRI techniques have been established during the last decades for identification of sequelae as well as for management decisions of pediatric TBI (124) |
| Glial fibrillary acidic protein (GFAP), S100 protein | GFAP and S100B protein are released as astroglial marker after pediatric TBI and correlate with TBI severity (127–129). |  |
| Myelin basic protein (MBP) | MBP is systemically increased after TBI in children (130) |  |
| Osteopontin (OPN) | OPN was found to be systemically enhanced in pediatric TBI correlating with TBI severity, intracranial lesions and mortality of the children (133). |  |
| Acute respiratory distress syndrome (ARDS) | | |
| Troponin | The cardiac specific marker troponin is also discussed as a reliable marker of lung contusion after pediatric trauma (9,150). | Besides a lower exposure to radiation, the X-ray imaging has much lower costs compared to CT scans. The CT should be considered if there are abnormalities in the initial diagnosis (147,148). This decision should be combined with a clinical evaluation and careful examination. |
| Surfactant-D | In adults, blunt chest trauma results in high serum levels of surfactant protein D (SP-D)(151). In a recent study, we observed a systemic increase of SP-D after hemorrhagic shock and cardiopulmonary resuscitation in newborn pigs (153). Translational studies need to be performed to evaluate the utility of SP-D in children. | In children, lung ultrasound is often used as a diagnostic tool for lung contusion (27,149) |
| Angiopoetin, soluble receptor for advanced glycation end products (sRAGE) | Ang2 and sRAGE were higher in survivors compared to non-survivors of pediatric ARDS. These biomarkers also correlate with the number of non-pulmonary organ failure in critically ill children (154,155). |  |
