# Supplementary material for: Laboratory Markers in the Management of Pediatric Polytrauma: Current Role and Areas of Future Research
Source: Front Pediatr. 2021 Mar 16;9:622753. doi: 10.3389/fped.2021.622753 (PMC8010656; doi:10.3389/fped.2021.622753)
Supplement: Supplemental Table 2 — Laboratory markers in the management of pediatric polytrauma. [file Table_2.DOCX]

| Organ System | Biomarker |
| --- | --- |
| Systemic Inflammation | C-reactive Protein (CRP) - routine diagnostics  Leukocyte number - routine diagnostics  Interleukin-6 (IL-6) - routine diagnostics  Interleukin-8 (IL-8) – rather experimental  Procalcitonin (PCT) - routine diagnostics |
| Acid-base equilibrium | Lactate- routine diagnostics |
| Coagulation | Prothrombin time- routine diagnostics  Partial thromboplastin time (pPTT) - routine diagnostics  Fibrinogen- routine diagnostics  Fibrin cleavage products - routine diagnostics  Thrombocyte number - routine diagnostics |
| Heart | Troponin I – routine diagnostics (TnT)  Heart fatty acid binding protein (HFABP) – rather experimental |
| Kidney | Creatinine - routine diagnostics  Neutrophil Gelatinase-associated Lipocalin (NGAL) – rather experimental  Kidney injury molecule-1 (KIM-1)- rather experimental  Cystatin C - routine diagnostics  IL-18 – rather experimental  Liver fatty acid binding protein (L-FABP) – rather experimental |
| Liver | Aspartate aminotransferase (AST) - routine diagnostics  Alanine aminotransferase (ALT) - routine diagnostics  Lactate dehydrogenase (LDH) - routine diagnostics |
| Brain | Angiopoietin-2 (AP-2) - rather experimental  Endothelin-1 (ET-1) - rather experimental  Endocan-2 (EC-2) - rather experimental  Neuron specific enolase (NSE) - routine diagnostics  Ubiquitin C-terminal hydrolase-L1 (UCH-L1) - rather experimental  Glial fibrillary acidic protein (GFAP) - rather experimental  S100B - rather experimental  Myelin basic protein (MBL) - rather experimental  Osteopontin (OPN) - rather experimental  High-mobility group box 1 protein (HMGB1) - rather experimental |
| Lung | Surfactant protein D (SP-D)- rather experimental  Angiopoietin-2 (AP-2) - rather experimental  Soluble receptor for advanced glycation end products (sRAGE) - rather experimental |
